# The use of a systems approach to increase NAD^+^ in human participants

**DOI:** 10.1038/s41514-023-00134-0

**Published:** 2024-02-01

**Authors:** John D. Henderson, Sophia N. Z. Quigley, Shruti S. Chachra, Nichola Conlon, Dianne Ford

**Affiliations:** 1 Department of Applied Sciences, Northumbria University, Northumberland Road, Newcastle upon Tyne, NE1 8ST UK; 2 Nuchido Ltd. Dissington Hall, Dalton, Northumberland NE18 0AD UK; 3 Present Address: Novo Nordisk Foundation Center for Basic Metabolic Research, University of Copenhagen, Blegdamsvej 3B, Mærsk Tårnet, 7, Sal, 2200 København N Denmark

**Keywords:** Biochemistry, Cell biology, Biomarkers

## Abstract

Reversal or mitigation against an age-related decline in NAD^+^ has likely benefits, and this premise has driven academic and commercial endeavour to develop dietary supplements that achieve this outcome. We used a systems-based approach to improve on current supplements by targeting multiple points in the NAD^+^ salvage pathway. In a double-blind, randomised, crossover trial, the supplement – Nuchido TIME+® (NT) - increased NAD^+^ concentration in whole blood. This was associated with an increase in SIRT1 and an increase in nicotinamide phosphoribosyltransferase (NAMPT) in peripheral blood mononucleocytes, lower concentrations of pro-inflammatory cytokines in plasma, including a reduction in interleukin 2 (IL2), a reduction in glycated serum protein and a shift in the glycosylation profile of immunoglobulin G (IgG) toward a younger biological age, all of which are likely to promote a healthier ageing trajectory.

## Introduction

Changes in many interrelated cellular processes and metabolic functions contribute to ageing. The key changes identified as underpinning cellular ageing are collectively known as the hallmarks of ageing^1^. Cellular senescence, mitochondrial dysfunction and deregulated nutrient sensing are among those studied most intensively, and chronically low NAD^+^ (nicotinamide adenine dinucleotide) has been identified as a central mediator of multiple hallmarks of ageing^2^. Interventions to prevent or reverse ageing-related changes in these variables, processes and pathways, or to stimulate counteracting cellular pathways, are made with the aim to slow or reverse the process of ageing and thus increase years of healthy life.

NAD^+^ is an attractive target point of intervention because, as the conduit of reducing power between the fundamental metabolic pathways of glycolysis, the TCA cycle and the mitochondrial electron transport chain, it plays a central role in the generation of cellular energy (ATP). Also, a sizable body of data provides evidence for an age-related decline in NAD^+^ levels in tissues including plasma and muscle, though this is not a universally consistent observation (reviewed in ref. ^3^). Also, NAD^+^ depletion is a feature of some diseases of accelerated ageing, including Ataxia Telangiectasia (AT), Xeroderma Pigmentosum group A (XPA), and Cockayne Syndrome (CS) (reviewed in ref. ^4^). NAD^+^ is also a cofactor for a number of enzymes, including enzymes with functions that impinge on cellular processes that have a role in ageing. Among these, the sirtuins are of likely particular importance. These enzymes, of which there are seven human members, are a family of deacylases and ADP-ribosyltransferases. SIRT1, the first named of the mammalian sirtuin family and the most extensively studied, catalyses the deacetylation of protein substrates at lysine residues in a reaction in which NAD^+^ is cleaved to release nicotinamide (NAM; see ref. ^5^ for recent review), and, like some other members of the family, catalyses the deacetylation of a range of substrates that have functions in a myriad of processes that impinge on ageing (see ref. ^6^ for a recent review). These include the transcription factor PGC1α (peroxisome proliferator-activated receptor-γ coactivator), which is activated as a result to stimulate mitochondrial biogenesis among other cellular processes, members of the FOXO family of transcription factors, resulting in downstream beneficial effects on glucose metabolism and insulin signalling, and NF-κβ, with consequent tempering of pro-inflammatory pathways^7^.

Among other enzymes that cleave NAD^+^ and appear to affect cellular ageing either directly or as a consequence of the knock-on effects of its consumption (for example reduced sirtuin action) are the PARPs (poly ADP-ribose polymerases) and CD38 (cluster of differentiation 38). PARP action involves cleavage of NAD^+^ at the N-glycosidic bond to generate ADP ribose. This is then added as a monomer or as a polymerised chain to proteins involved in a number of cellular functions, which include the response to DNA damage by base excision repair of single strand breaks^8^. PARP activity has been associated correlatively with longer lifespan and slower ageing, which is likely attributable in part to this role in the DNA damage response and thus to genome stability (e.g., ref. ^9^). However, PARP activity is something of a double-edged sword because the consumption of NAD^+^ reduces availability for the generation of ATP and for the action of enzymes, including the sirtuins, that afford protection against ageing.

CD38 has been described as the principal NAD^+^ hydrolase in the cell. It has been shown to have multiple functions, among which is a role in cell signalling by generating, from NAD^+^, the second messenger molecule, cyclic ADP ribose (cADPR). cADPR plays a role in intracellular Ca^2+^ signalling through activation of ryanodine receptors to mobilise Ca^2+^ from the endoplasmic reticulum and also has a role in multiple aspects of the inflammatory response^10^. The protection against features of ageing afforded by the pharmacological inhibition or knockout of CD38 in mice, such as protection against obesity^11^, improved glucose tolerance, muscle function, exercise capacity and cardiac function^12^ and increased lifespan^13^, have been attributed to the NAD^+^-sparing effect of these interventions.

**Fig. 1:**
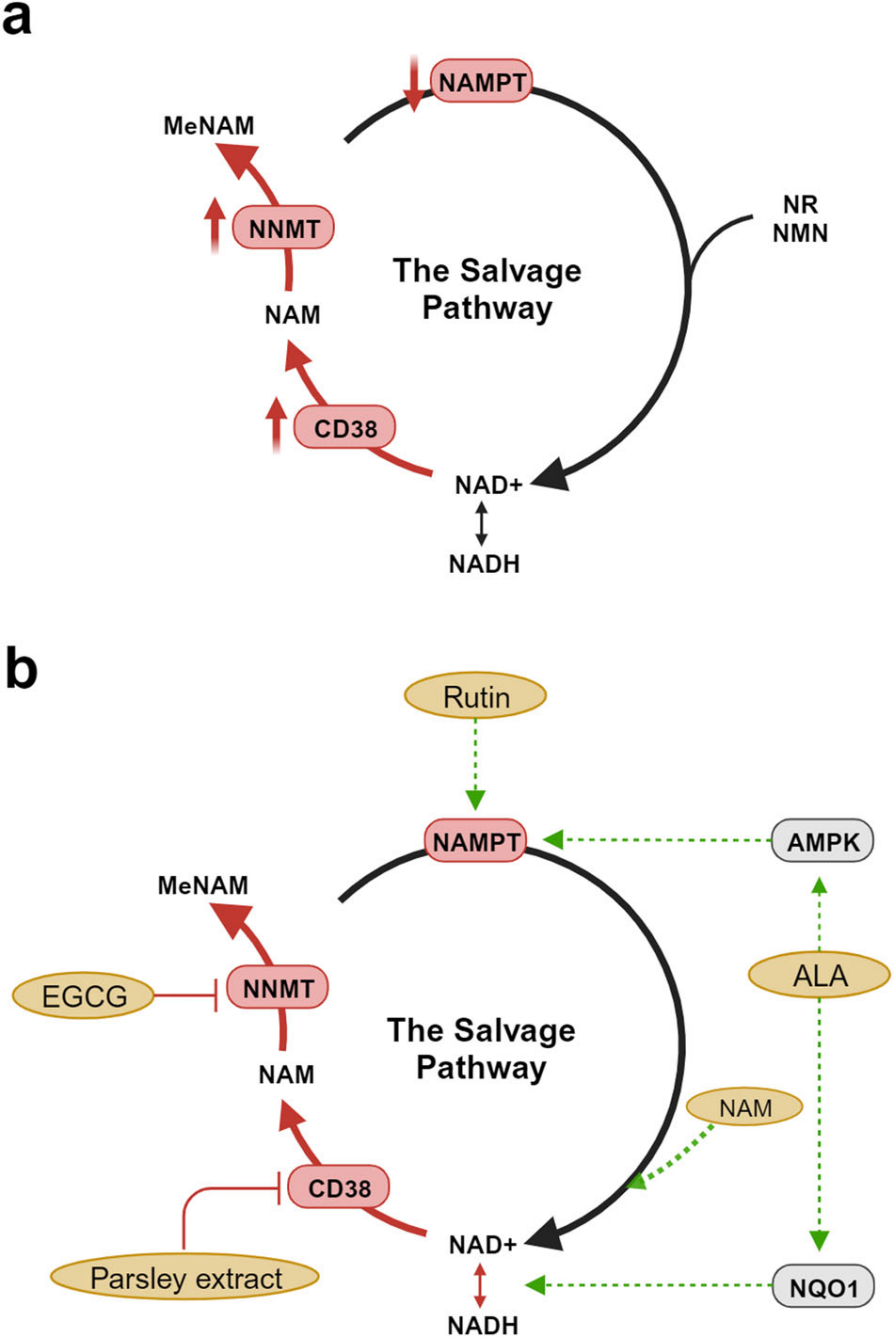
Schematic representations of the salvage pathway for NAD^+^ generation. **a** shows impairment of the pathway by age-associated changes. **b** shows points of redress through a multitarget approach that forms the basis of the NT dietary supplement. **a** CD38 becomes overexpressed during ageing due to chronic activation from persistent low level ‘inflammaging’. CD38 cleaves NAD^+^ to produce nicotinamide (NAM). Increased consumption of NAD^+^ by CD38 leads to an accumulation of cellular NAM. Aged cells exhibit lower levels of NAMPT, the key rate-limiting enzyme for adequate NAD^+^ recycling from NAM, resulting in reduced NAD^+^ production and an accumulation of NAM. NNMT is recruited to methylate and excrete excess NAM as methyl nicotinamide (MeNAM). **b** The NT dietary supplement contains multiple ingredients to target the root causes of NAD^+^ decline. Rutin and alpha lipoic acid (ALA) act to increase expression of the rate-limiting enzyme NAMPT. ALA induces NAMPT expression indirectly by activating the cellular energy sensor AMPK which is known to increase NAMPT expression. ALA also increases expression of NQO1 which increases NAD^+^ by oxidation of NADH, pushing the NAD^+^:NADH ratio in a more favourable direction. Parsley extract contains a high proportion of natural apigenin which acts to inhibit CD38. EGCG acts to inhibit NNMT to promote recycling of NAM into NAD^+^ by the Salvage pathway rather than its methylation and excretion. NAM is used as an NAD^+^ precursor as it is the most bioavailable of all the NAD^+^ precursors.

There is currently much interest academically and commercially in approaches to boost NAD^+^ as a potential anti-ageing therapy. In vivo NAD^+^ restoration has been investigated extensively and demonstrates whole body benefits, recently reviewed in detail^14^. The instability and low bioavailability to most cell types of NAD^+^ render direct oral supplementation inefficacious. Dietary supplements intended to increase NAD^+^ in human subjects feed in at points in the process of biosynthesis, of which there are three pathways (see ref. ^4^ for a full description). Of these, the salvage pathway is the dominant pathway. This pathway recycles NAM released by the cleavage of NAD^+^ through the action of NAD^+^-consuming enzymes including the sirtuins, PARPs and CD38. Most dietary supplements are based on providing a supply of precursors to the salvage pathway, most commonly NR or NMN (nicotinamide mononucleotide). We summarise the outcomes of 10 we identified, which all provided NR, in a recent review article^4^. Of these, seven measured NAD^+^ in blood (whole blood or peripheral blood mononucleocytes (PBMCs)) and three measured NAD^+^ in skeletal muscle. Significant but variable increases in NAD^+^ are reported. Modest increases (40–59%) were achieved using the dose of 300 mg/d approved by the FDA and EFSA, with larger increases (more than a doubling of the sample mean or doubling of concentration in the highest responder) requiring doses far in excess (1000–2000 mg/d). Even at these very high doses, the three studies that measured NAD^+^ in skeletal muscle detected no increase^15–17^. Nine published studies that provided NMN report similarly variable outcomes. Increases in NAD^+^ in whole blood, plasma, serum or PBMCs were measured in six of these^18–23^, which include a study that achieved a 5.7-fold mean increase after 60 d at 600 mg/d^23^, but three achieved no increase, despite use of similar doses^24–26^. Factors such as duration of the trial period, participant demographics, physical properties of NR or NMN administered (e.g., particle size and crystalline form), methods of sample collection and NAD^+^ measurement likely account for some of this variability. However, we argue that a key factor that limits efficacy is likely to be changes to the NAD^+^-metabolising machinery in the cell that occur as the cell gets older, which themselves curtail the ability of the cell to generate NAD^+^ from NR, NMN and other precursors or to consume NAD^+^ at a higher rate. Notably, the enzyme nicotinamide phosphoribosyltransferase (NAMPT), which catalyses the rate-limiting step in the NAD^+^ salvage pathway, has been shown to decline with age in mice and rats^27–33^, as well as humans^27,34^. There is good evidence that CD38, the major cellular NAD^+^ hydrolase and mediator of various aspects of the inflammatory response, becomes overexpressed during ageing, accompanying the high level of inflammation frequently reported in older tissues (referred to as ‘inflammaging’)^35,36^. Increased expression of the methylating enzyme nicotinamide-N-methyltransferase (NNMT) also leads to the methylation and excretion of NAM as methyl nicotinamide (MeNAM) further limiting the recycling of NAM to NAD^+^ by the salvage pathway^37^. We argue that a significant limitation of simple, single precursor supplements is that, while providing a supply of substrate into the salvage pathway, this cannot be optimally converted to NAD^+^ because of the aforementioned issues that prevent optimal NAD^+^ conversion and usage. Figure 1a shows the age-related changes to the NAD^+^ salvage pathway and enzymes that contribute to depletion of NAD^+^. Thus, we took a systems approach to administer participants with a supplement (NT) targeting multiple enzymes involved in NAD+ metabolism whilst also providing NAD^+^ precursor (Fig. 1b). Table 1 lists the components of NT and specifies the basis (intended action) for including them in the supplement^38–50^.

**Table 1:** Components of NT and target points of interaction with the cellular processes that influence cellular NAD^+^ content.

Component	Typical content in daily dose (6 capsules)	Intended action
Nicotinamide	500 mg	Substrate for NAD^+^ synthesis (salvage pathway)
Alpha lipoic acid	600 mg	Activate NAMPT through activation of AMPK^38,39^; activate NQO1 to promote oxidation of NADH to NAD^+^^40^, reduce production of free radicals^41^; antioxidant^42^
Zinc	10 mg	Antioxidant; support immune function^43,44^
Vitamin C	20 mg	
Botanical blend comprising:	2110 mg	
Green tea extract (providing Epigallocatechin gallate (EGCG) and other catechins)		Reduce expression of NNMT^45^, which methylates NAM to excreted product MeNAM^46^.
Rutin (providing Quercetin and Troxerutin)		Increase activity of NAMPT (rate-limiting enzyme in salvage pathway)^47,48^;
Parsley leaf extract (providing apigenin)		Inhibits CD38^49^ (primary NAD+ hydrolase)
Black pepper fruit extract		To enhance oral bioavailability of phytonutrients^50^

## Results

**Fig. 2:**
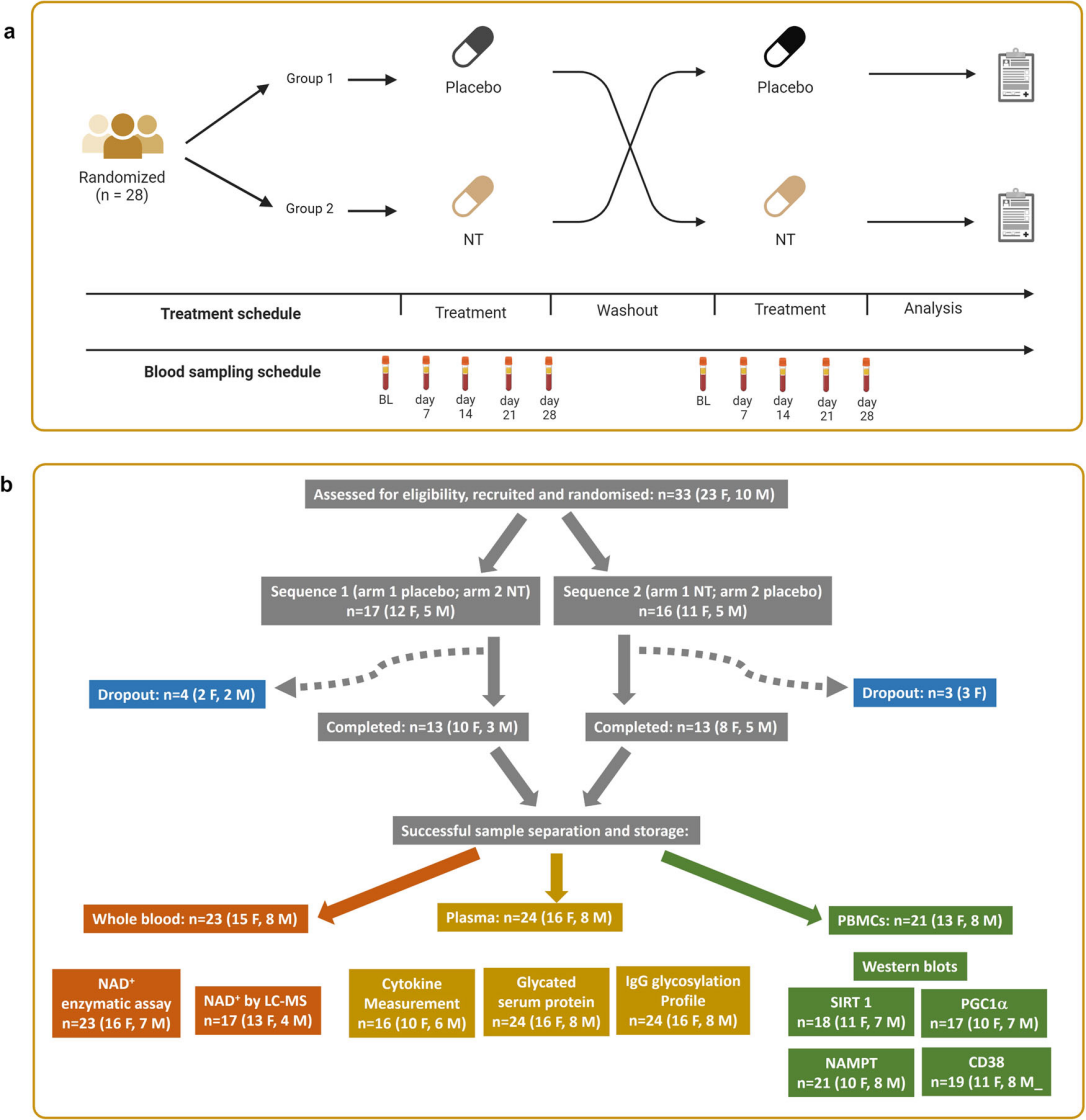
A trial to test the efficacy of NT to elevate NAD^+^ in the blood of human participants. **a** Double-blind, crossover, placebo-controlled trial design. (Created using BioRender). **b** CONSORT diagram showing the flow of participants through the trial and the inclusion of samples of blood and its fractions in the different assays conducted.

We conducted a double-blind placebo-controlled trial (Fig. 2) to determine if the dietary supplement NT (Nuchido TIME+®) designed using a systems approach to address the root causes of NAD^+^ decline, was efficacious in increasing the NAD^+^ concentration in whole blood. We recruited 33 human participants, spread across an age range of 20–80 y, of whom 26 (18 female) completed the study. One drop out, before attending, was due to personal reasons and three failed to attend to provide blood samples. One participant withdrew at the first visit to provide a baseline sample (arm 1) and another withdrew at the beginning of arm 2. We were unable to obtain a blood sample at the beginning of arm 2 for the sixth non-completer. All participants were asked about adverse effects. Very few were reported; two participants reported trouble sleeping when taking NT, and one reported stomach irritation. One participant reported feeling hyperactive on the placebo arm. No other adverse effects were reported.

### Toxicity

The activity of alanine aminotransferase, the activity of alkaline phosphatase, and the concentration of albumin in plasma were unaffected by either NT or the placebo (Supplementary Table 1). Thus, we found no indication that the supplement induced any level of hepatic toxicity.

### Effect of NT on blood NAD^+^, NAM and NMN concentration

**Fig. 3:**
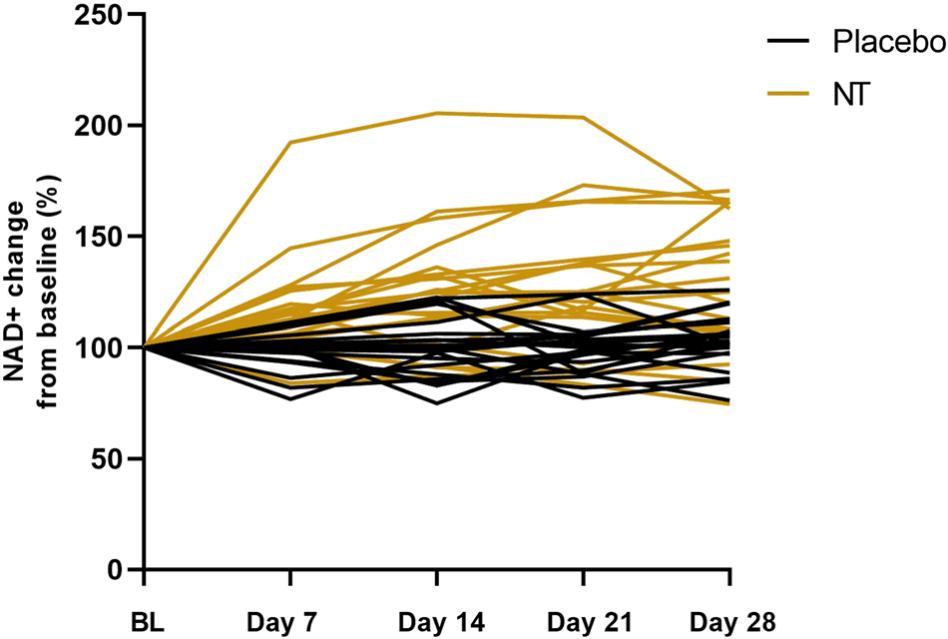
Measures of NAD^+^ in whole blood for participants (*n* = 23) who completed the trial. Values are plotted separately for each participant, colour-coded as shown in the key for each arm of the trial, as % change from baseline at each of the 4 sampling points.

**Fig. 4:**
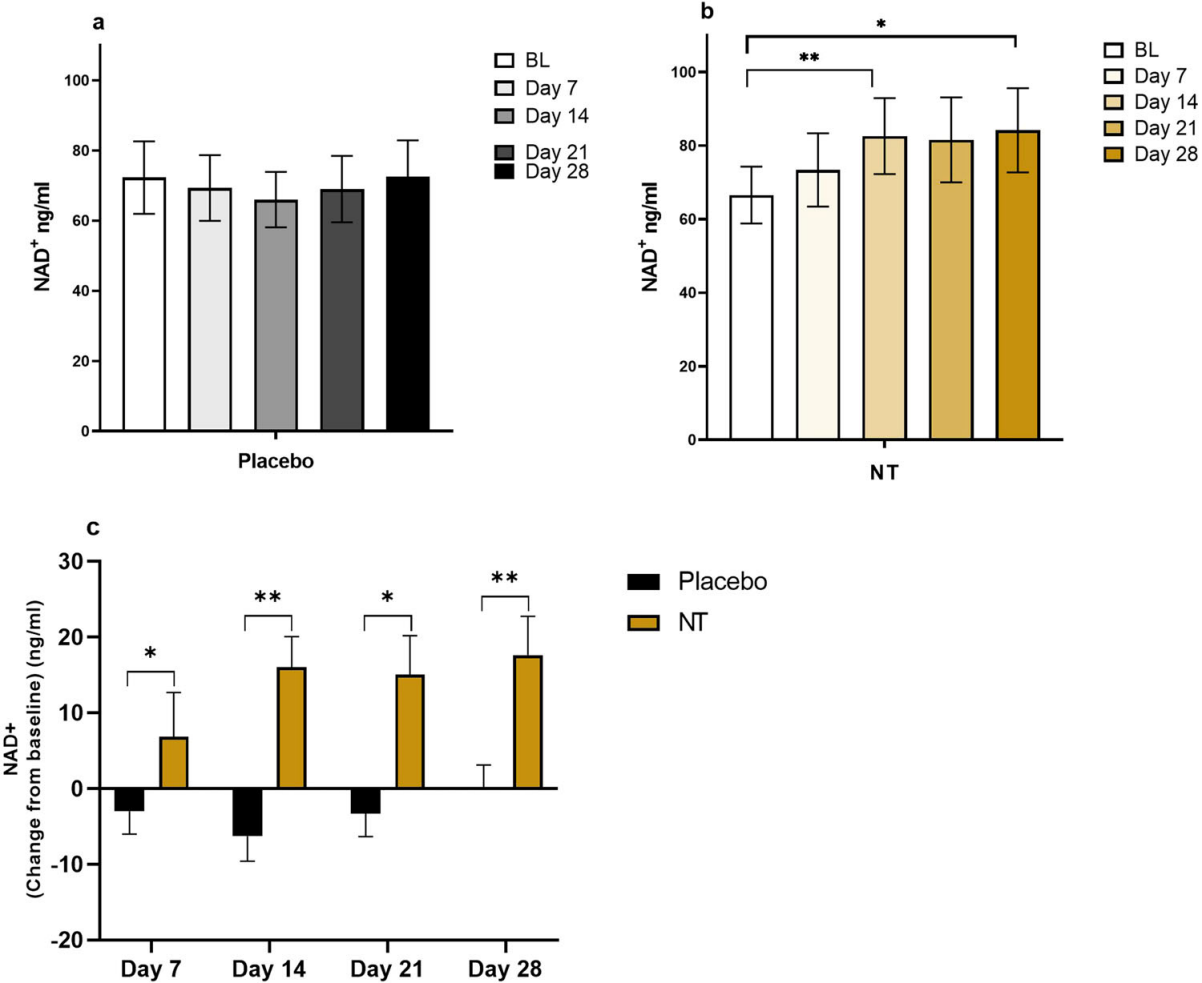
Concentration of NAD^+^ in whole blood, measured using the NAD^+^/NADH-Glo Assay (Promega), for participants (*n* = 23) who completed the trial. Values are plotted as measured concentrations (mean ± SEM) (**a** and **b**) or as change from baseline (mean ± SEM) (**c**). **b** *P < 0.05; **P < 0.01 by repeated measures 2-way ANOVA, followed by Bonferroni post-hoc test. **c** *P < 0.05; **P < 0.01 by Student’s paired *t*-test.

Figure 3 shows the profile of NAD^+^ concentration measured using a commercial fluorometric assay in whole blood over each arm of the trial period for each participant. Two-way, repeated measures ANOVA showed a significant main effect of NT with time on NAD^+^ concentration (*P* = 0.007), and Bonferroni post-hoc testing showed a significant increase compared with baseline (mean 66.55 ng/ml; SD 36.97) at weeks 2 (mean 82.59 ng/ml; SD 49.56, *P* = 0.008) and 4 (mean 84.17 ng/ml; SD 54.82, *P* = 0.029; Fig. 4b). These data show that NT increased NAD^+^ in whole blood at week 4 by an average (mean) of 26.5%. However, as is seen from Fig. 3, there was considerable inter-individual variability in response size. In the three individuals where the response was greatest, increases of 105%, 73% and 65% were achieved. There was no significant effect of the placebo (Fig. 4a). Analysis of the data at each time point by Student’s paired *t*-test showed that the concentration of NAD^+^ in whole blood was significantly greater on NT than on placebo at all points measured (Fig. 4c).

**Fig. 5:**
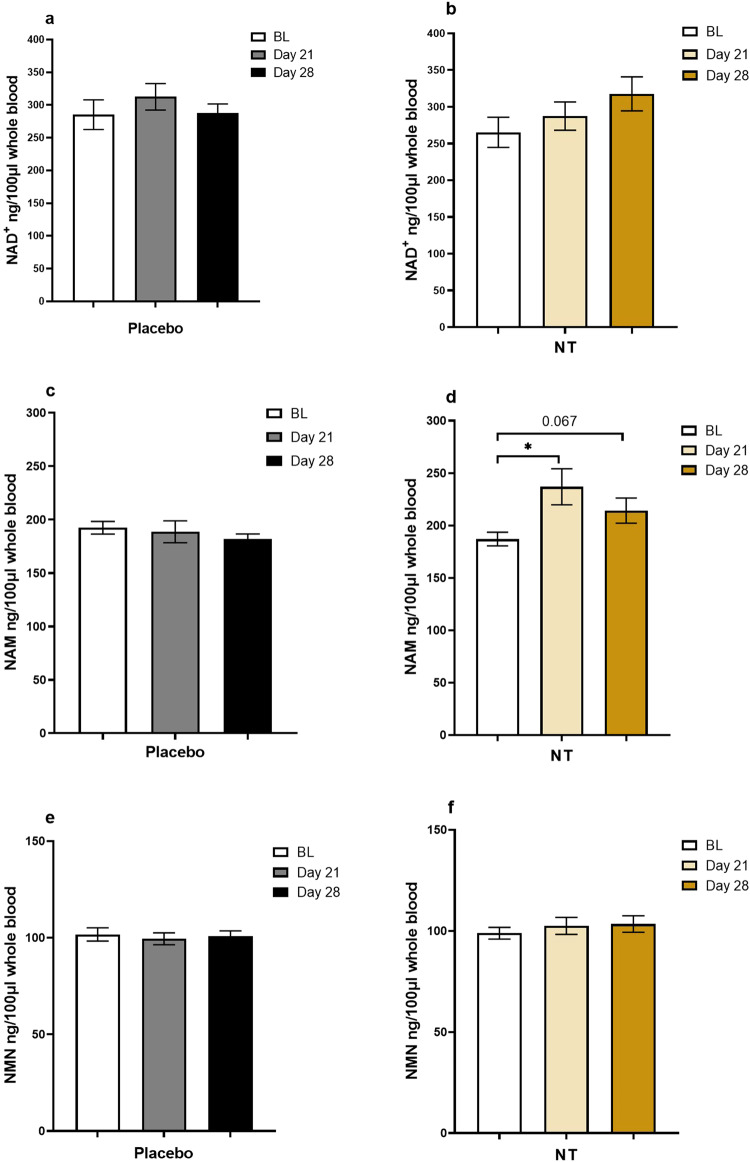
Concentration of NAD^+^, NAM and NMN in whole blood, measured by LC–MS. **a**, **c**, **e** Placebo; **b**, **d**, **f** NT. Values are mean ± SEM; *n* = 17. *P < 0.05 by repeated measures 2-way ANOVA, followed by Bonferroni post-hoc test.

We also measured NAD^+^ in whole blood, along with NAM and NMN, in 17 of the participants at baseline, 21 days and 28 days by LC–MS. Use of the subsample was a pragmatic measure enforced by the availability of sufficient remaining sample following measurement using the fluorometric assay. These data revealed the same trend (compare Fig. 5a) (placebo) with Fig. 5b (NT) but this did not reach statistical significance when data were analysed by 2-way repeated measures ANOVA. To determine if the results of the two different measurement techniques were discordant, or if the reduced power of the analysis accounted for the loss of a significant effect of NT on NAD^+^ measured by LC–MS, the equivalent reduced dataset (same 17 participants, baseline, 21 days and 28 days only) obtained using the commercial fluorometric kit were re-analysed by 2-way repeated measures ANOVA. This analysis did not reveal a significant effect of either NT or placebo on NAD^+^. Thus, the reduced power of the analysis, due to the smaller sample set, may have obscured a true significant effect of NT to increase NAD^+^ in whole blood, which was detectable with the larger sample. However, at the point of making the measurements by LC–MS, samples had been stored at −80 °C for several months due to interruption of work by the COVID-19 pandemic, meaning that NAD^+^ degradation may also have a contributory factor. Moreover, as can be seen from Fig. 3, four study participants showed a notably large increase in blood NAD^+^ concentration across the sampling time points measured using the fluorometric enzymatic assay, and one showed an increase at day 28 that approximated the increase of the other four high responders at this point. Of these participants (numbers 11, 17, 23, 26; 18 (late responder), Supplementary Table 1) only two of the four high responders (numbers 11, 23) plus the late responder (number 18) were included in the group for which measurements of NAD^+^ were made by LC/MS, which will have influenced the statistical analysis.

Analysis by one-way repeated measures ANOVA showed an effect of NT but not the placebo, on NAM (Fig. 5c, d). This was revealed by Bonferroni post-hoc testing to be a significant (*P* = 0.016) increase in the mean concentration compared with baseline (mean 186.96 ng/100 μl, SD 27.60) at 21 days (mean 236.88 ng/100 μl; SD 73.22). There was a trend (*P* = 0.067) towards an increase also at 28 days (mean 214.19 ng/100 μl; SD 50.90). There was no significant change in NMN (Fig. 5e, f).

### Effect of NT on expression of selected proteins

**Fig. 6:**
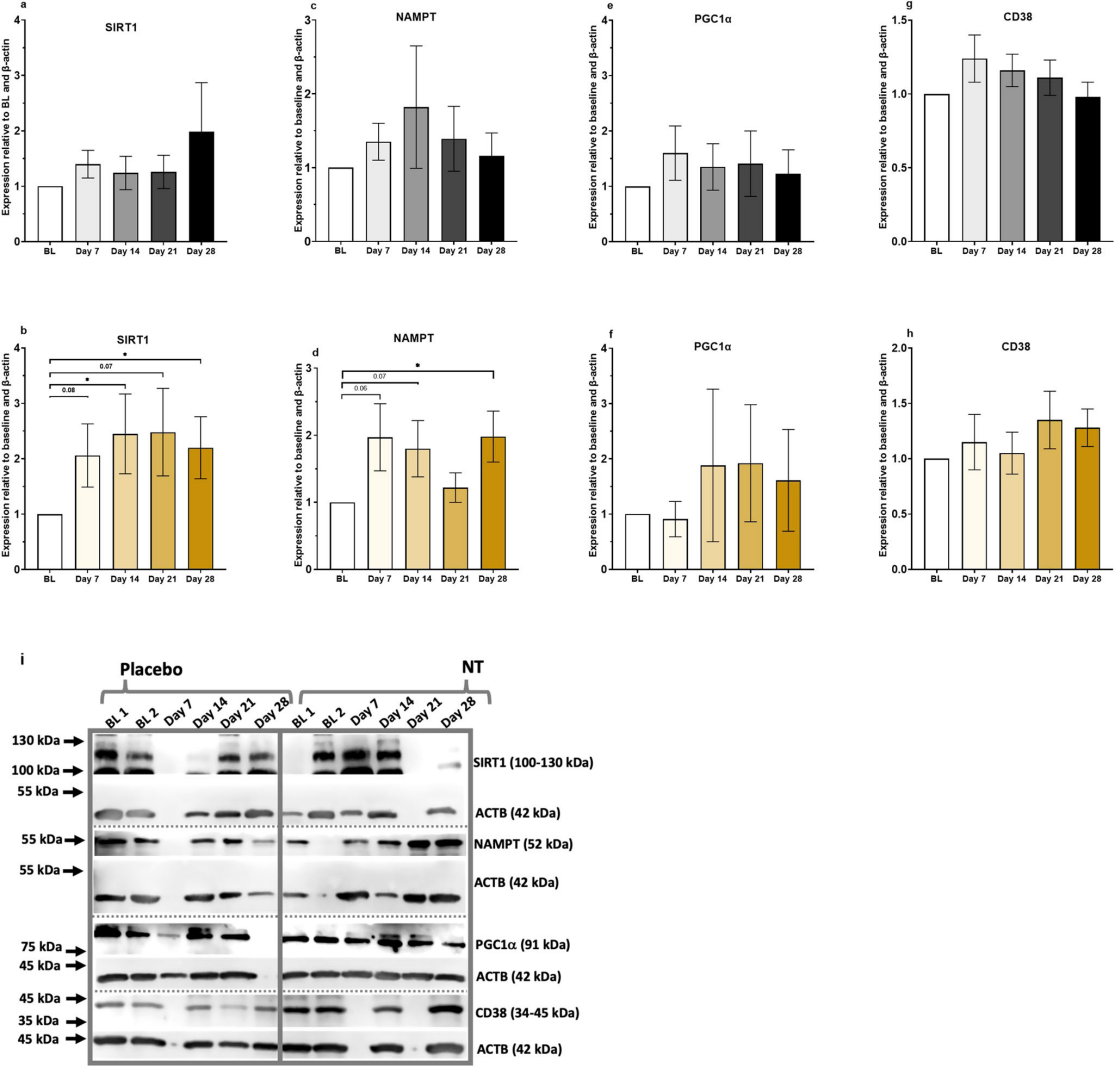
Relative quantities of SIRT1, NAMPT, PGC1α, and CD38 protein in PBMCs in trial participants measured by western blotting. **a**, **c**, **e**, **g** Placebo. **b**, **d**, **f**, **h** NT. Data are expressed as mean ± SEM; *n* = 18–21 and are relative to corresponding signal for ACTB and normalised to the measurement at baseline, which was used and is plotted as the mean of the two baseline samples (BL1 and BL2) collected at the same time of day in the week preceding the intervention. Gels for each primary antibody used derive from the same experiment and were processed in parallel. The four participants who showed the highest increases in whole blood NAD^+^ concentration (numbers 11, 17, 23, 26; Fig. 3 and Supplementary Table 1), as well as the late responder (number 18), were included in all western blot assays. *P < 0.05 by Student’s *t*-test. **i** Representative western blots for each antibody, as labelled, with corresponding signals for ACTB.

Our trial was designed primarily to determine if NT was efficacious in increasing NAD^+^ in whole blood. However, we measured a number of other secondary outcome variables to explore if there was any evidence of beneficial consequent (or parallel) effects. Thus, we measured SIRT1, NAMPT, PGC1α and CD38 in PBMCs by western blotting at all sample time points in 18–21 participants for each antibody (as determined by sample availability). The four participants who showed the highest increases in whole blood NAD^+^ concentration (numbers 11, 17, 23, 26; Fig. 3 and Supplementary Table 1), as well as the late responder (number 18), were included in all western blot assays. For these measurements, signal intensities were expressed relative to the signal for ACTB. To enable combination of data across blots, these ratios were then normalised against the mean ratio value for the two baseline measurements. This analysis showed that NT increased SIRT1 significantly (*P* < 0.05) at 14 and 28 days (Fig. 6a, b) and increased NAMPT (*P* < 0.05) at 28 days (Fig. 6c, d). We measured no changes in PGC1α or CD38 (Fig. 6e–h). Figure 6I shows examples of blots for each protein antigen. Uncropped blots for SIRT1 and NAMPT, and corresponding ACTB loading controls are provided as Supplementary Figure 2.

### Effect of NT on expression of cytokines

**Fig. 7:**
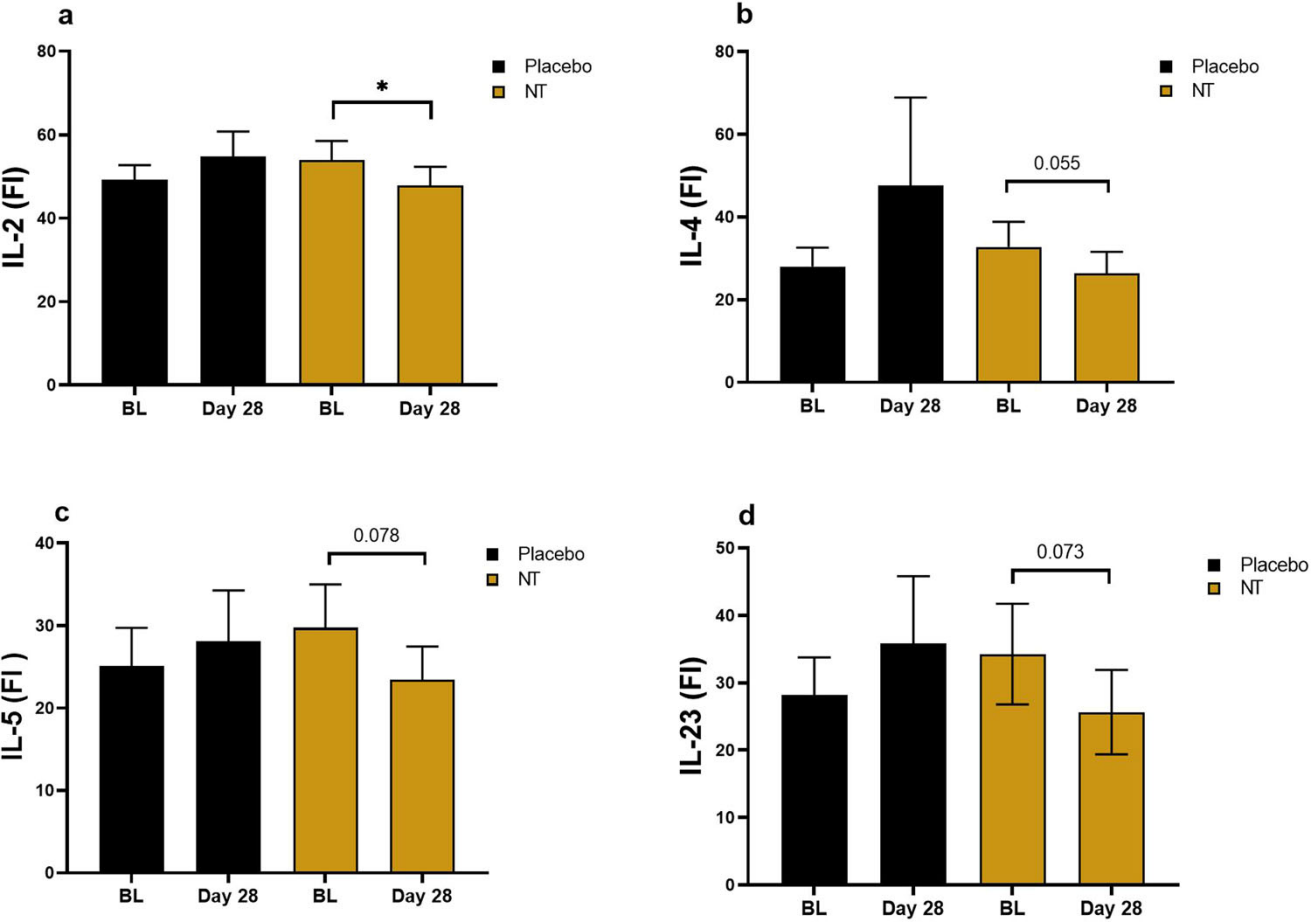
Concentration of selected cytokines in plasma of trial participants. **a** IL2; **b** IL4; **c** IL5; **d** IL23. Data are expressed as mean ± SEM as relative measures of assay signal (fluorescence intensity); *n* = 16. The four participants who showed the highest increases in whole blood NAD^+^ concentration (numbers 11, 17, 23, 26; Fig. 3 and Supplementary Table 1), as well as the late responder (number 18), were included in all *P < 0.05 by Student’s paired *t*-test.

We also measured a panel of 14 cytokines at baseline and 28 days in the plasma of 16 participants (Fig. 7) and detected a reduction (*P* < 0.05) in the mean value compared with baseline in IL2 after 28 days on NT. Lower mean values at 28 days on NT compared with baseline for IL4, IL5 and IL23 were also close to reaching statistical significance (*P* = 0.055, 0.079 and 0.073, respectively). In contrast, there were no changes in any of the cytokines measured after 28 days on the placebo (*P* > 0.28 for every cytokine). The four participants who showed the highest increases in whole blood NAD^+^ concentration (numbers 11, 17, 23, 26; Fig. 3 and Supplementary Table 1), as well as the late responder (number 18), were included in the group of participants in whose samples we measured cytokines.

### Effect of NT on biological age

**Fig. 8:**
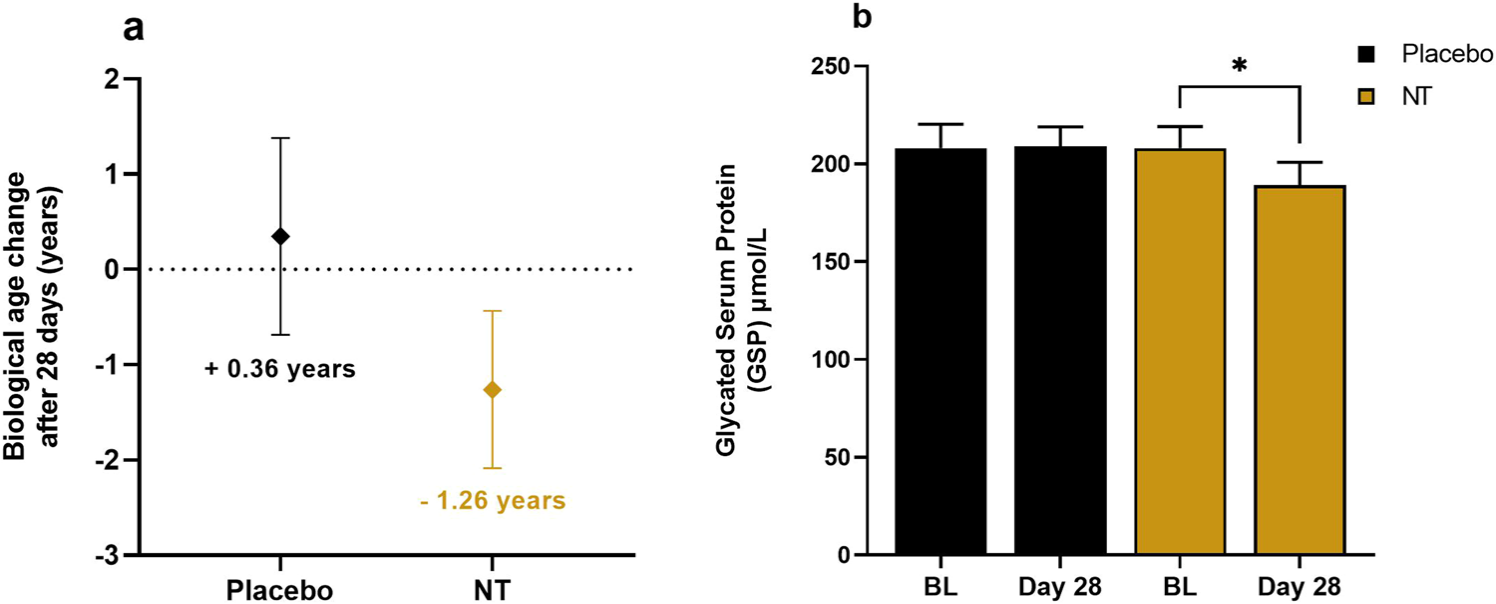
IgG glycosylation and serum protein glycation in trial participants. **a** Changes to IgG glycosylation profiles measured then extrapolated as ‘biological age’ through commercial service (GlycanAge, UK) expressed as difference from baseline at 28 d; mean with 95% CI (*n* = 24). **b** Serum protein glycation (Jinfiniti, Augusta, USA) at baseline and 28 d expressed as mean ± SEM (*n* = 24). *P < 0.05 by Student’s *t*-test.

Measurement of changes to IgG glycosylation patterns, as an indicator of ‘biological age’, revealed an average difference between baseline and 28 days of +0.36 y on placebo and −1.26 y on NT (Fig. 8a).

### Effect of NT on serum protein glycation

Glycation of serum protein was also measured and found to be reduced on NT, but not placebo (*P* < 0.05) (Fig. 8b).

## Discussion

We aimed to improve on current approaches to increasing systemic NAD^+^ levels by formulating a dietary supplement (NT) based on targeting multiple points in the network of NAD^+^ metabolism. We provided the substrate for NAD^+^ synthesis though the salvage pathway as NAM, a compound present in a normal diet. Uniquely, we aimed to overcome limitations with current approaches that fail to address the root causes of NAD^+^ decline, namely a reported age-related decline in NAMPT, the rate-limiting enzyme essential for endogenous NAD^+^ recycling^4^. Our approach to achieving this was to include in NT alpha lipoic acid and rutin, both of which have been reported to activate NAMPT^38,47^. Significantly, a measured increase in the expression of NAMPT in PBMCs of participants while taking NT, but not placebo, demonstrated the efficacy of the supplement to achieve this key, target outcome.

Our trial was afforded particular statistical power by being of crossover design. Compared with placebo, NT increased NAD^+^ concentration in whole blood at each of 4 weekly sampling time points. Also, changes in established ageing-related markers, including SIRT1 protein in PBMCs and pro-inflammatory cytokines in plasma^1,51^ in a direction favourable to slower ageing were observed in parallel.

Participants in this study spanned an age range of 21–72 y. While not designed to test an effect of age on response, we nonetheless analysed the data for any evidence that age had any effect on the increase in whole blood NAD^+^ concentration achieved. We found no statistically significant correlation between participant age and the size of the increase in NAD^+^ in response to NT (*R* = 1.64; *p* = 0.229 by Spearman’s rank correlation). Also, analysis by Student’s unpaired *t*-test does not show that the age profile of the 4 participants who showed the biggest increases in whole blood NAD^+^ concentration (40 y, 59 y, 61 y, 66 y -mean 56.5 ± 11.4 years (mean ± SD)) differs significantly from that of the other participants (46 ± 15.0 years (mean ± SD)) (*p* = 0.20).

Variations between studies in daily precursor dose and other features mean that direct comparison with increases achieved, or not, in other studies - which have yielded inconsistent results - has limitations.

To further increase efficacy through counteracting age-related changes in NAD^+^ metabolism in the cell, we included agents aimed to simultaneously reduce NAD^+^ breakdown by the NAD^+^ hydrolase CD38. The observed reduction in pro-inflammatory cytokines would be commensurate with a reduction in CD38 activity, as CD38 has been shown to facilitate signalling pathways that lead to the production of pro-inflammatory cytokines^52^. NT includes parsley leaf extract as a natural source of apigenin, a known inhibitor of CD38^49^, with the aim of achieving this benefit. An alternative or additional contributory factor to the reduction in pro-inflammatory cytokines could be increased SIRT1 activity, which has been shown to have this effect^53^.

After taking NT, there was also an average reduction in ‘biological age’ across the study participants of 1.26 y, measured as a change to IgG glycosylation patterns, which has been identified as a biomarker and developed into a commercial assay by GlycanAge. In large population cohorts, changes to the IgG glycome have been found to be a highly effective indicator of an individual’s biological age, lifestyle and risk of age-related disease^54,55^. Furthermore, the plasticity of IgG glycosylation makes this a biomarker that is responsive to intervention.

The measured reduction in serum protein glycation is also indicative of an improvement in glucose tolerance elicited by NT. Low NAD^+^ is associated with metabolic dysfunction, which can be attenuated by an NAD^+^-dependent increase in SIRT1 activity^56^. Indeed, SIRT1 activation is a major target of interest for diabetic neuropathy, which is associated with high levels of serum protein glycation^57,58^.

In addition to the primary reason for which components of the supplement were selected - reported action at specific points in the cellular processes that affect NAD^+^ levels - some also have antioxidant and other actions that could potentially augment any efficacy of the supplement to slow the ageing process. Specifically, the catechins, included primarily as inhibitors of NNMT, are powerful antioxidants^59^, as is the polyphenol troxerutin^60^, which was incorporated with the aim that its reported action to increase NAMPT expression would stimulate the salvage pathway of NAD^+^ biosynthesis^47,48^. Moreover, an added potential, incidental benefit of deriving apigenin, EGCG and quercetin - intended to influence cellular NAD^+^ content - from the natural extracts of parsley leaf, green tea leaf and rutin respectively (included in the supplement as a mixed ‘botanical blend’ of extracts), is that these are also rich in additional natural antioxidants and polyphenols. Further potential benefits of these components include stimulation of the removal of senescent cells (senolysis) by quercetin^61^, and suppression by rutin of inflammation associated with cellular senescence (senomorphic activity)^62^.

Comparison of baseline NAD^+^ concentration in blood before each arm of the trial in those participants randomised to take NT in arm 1 and placebo in arm 2 did not reveal a statistically significant difference, indicating that the 28-day washout period was sufficient and also that the elevation achieved was transient. However, this does not exclude the possibility that other effects may persist - in particular epigenetic effects as a result of the activation of sirtuins leading to histone deacetylation. It would be of interest in further work to measure such potential epigenetic changes.

Further to the increase in SIRT1, reduction in pro-inflammatory cytokines and favourable changes to serum protein glycation and biological age observed in the current study, we aim in future work to investigate if these changes and the increase in NAD^+^ we measured translate to more tangible physiological changes of more obvious benefit as individuals get older. In this work, we will focus in particular on the skin as there is growing evidence that NAD^+^ plays a critical role in the biology of skin ageing^63^. Key variables to measure will be skin appearance, including levels of wrinkling, pigmentation, and elasticity. Although arguably of cosmetic importance, the changes in these measures that accompany ageing are reflective of a decline in barrier function, thinning of the dermis and epidermis, and an increase in fragility and susceptibility to sunburn. Maintenance of barrier function is important to reduce water loss and in thermoregulation. Skin resilience to physical damage from trauma and sunburn is important to reduce the likelihood of skin injury, which in older people particularly can lead to infection. Moreover, barrier immunity is reduced in aged skin, which in part appears to contribute to the increase in skin cancer observed in older people^64^.

Limitations of the study in relation to determining efficacy of NT to increase NAD^+^ in blood include that a single sample was taken from each participant to make each of the two baseline measurements and at each of the four weekly visits during each arm of the intervention. NAD^+^ concentrations show circadian fluctuation^65^, and it was not pragmatic to take samples from every participant at the same point in the day. Thus, there is likely to have been a level of noise in the data relating to this variability. However, our crossover design afforded the opportunity to counteract this variability with regard to statistical analysis, to an extent, by having each participant attend to provide samples at times in the day as closely aligned as possible for that individual. Another limitation is the relatively short treatment period. It would be optimal to measure the effects of NT over many months, and whether the increases in NAD+ and secondary outcomes (cytokine profile, protein glycation, IgG glycosylation) are sustained, enhanced or reduced during long-term supplementation. For the purpose of this study however it was not practical to conduct the study over such a long period.

In summary, this intervention study demonstrates efficacy of a dietary supplement designed using a systems approach to address the root causes of NAD^+^ decline to increase systemic NAD^+^ levels and indicates that co-administered components of the supplement contribute to this positive effect. Furthermore, we report a positive change in multiple healthy-ageing biomarkers (besides NAD^+^) in response to an NAD^+^ targeting supplement in a human cohort. Hence, targeting multiple points in the complex network of NAD^+^ metabolism represents a promising strategy for the new development of new ‘healthy-ageing’ interventions.

## Methods

### Human intervention study

Ethical approval for the study – a double-blind, randomised crossover trial – was granted by the Northumbria University Research Ethics Committee (reference 16845). All subjects recruited provided written informed consent to participate in the study.

Thirty-three human participants, age 20–80 y (8 male), were recruited in September 2019. Measurements of NAD^+^ in whole blood after the consumption of NT by 2 human subjects in a preliminary test gave an average effect size of 1.3 SD of mean, detection of which at 80% power and *P* < 0.05 (2 tailed) would require 12 participants, and for which the 33 participants provided 99% power.

Volunteers were excluded from participation on the basis of meeting any of the following criteria: Regular consumption of supplements containing niacin (vitamin B3), niacinamide nicotinamide riboside or nicotinamide mononucleotide; Food allergies or sensitivities; Currently taking blood pressure medication; Currently taking blood thinning medication (e.g. aspirin, warfarin, heparin); History or current diagnosis of drug/alcohol abuse; History of kidney or liver disease, or other severe diseases of the gastrointestinal; History of neurological or psychiatric illness (excluding depressive illness and anxiety); Blood disorders (e.g., anaemia, haemophilia, thrombocytosis); Heart disorder, or vascular illness; Chronic gastrointestinal problems (e.g., Inflammatory Bowel Disease, Irritable Bowel Syndrome, coeliac disease); Any known active infection; Diagnosed with or may be at risk of having syphilis, hepatitis, the Human T-lymphotropic virus, the Human Immunodeficiency Virus or any other infectious blood-borne disease; Health condition that would prevent fulfilment of the study requirements; Currently participating in or in the past 3 weeks participated in other clinical or nutrition intervention studies. For each arm of the trial, each participant attended on 2 separate occasions at the same time of day in the week preceding administration of the test intervention to provide a baseline sample of blood. NT or placebo (microcrystalline cellulose) was taken as a daily dose of 6 capsules – 3 with breakfast and 3 with lunch - for a period of 28 days. Participants were asked to keep their weekly diet and sleep patterns as constant as possible and to avoid any drinks or supplements containing niacin, nicotinamide or nicotinamide riboside. Participants attended on days 7, 14, 21 and 28 of the intervention period to provide a blood sample, collected as for the baseline samples. Each arm of the trial was separated by a washout period of 28 days. Figure 2 shows the study design.

The coding used to mark the active supplement (NT) and placebo (microcrystalline cellulose) was revealed only after the intervention trial, measurement of samples and statistical analysis was complete.

### Blood collection, preparation and storage

Blood (2 × 8 ml) was collected via venepuncture from the basilic, cephalic or median cubital vein into a vacutainer (Sodium heparin, BD), which was inverted 2–3 times then placed on ice. Two millilitres of whole blood was then immediately transferred to storage at −80 °C in a cryovial and PBMCs and plasma were prepared from the remainder of the sample. For this, all steps were performed on ice, except centrifugation (3 °C) and completed within 1 h of blood collection. The sample was diluted 3-fold in 2% FBS/1 × PBS then added on top of 15 ml Lymphoprep™ (STEM CELL Technologies) in a 50 ml Falcon tube. This was then centrifuged at 1000 × *g*, for 12 min at 3 °C. The upper plasma layer was then removed using a Pasteur pipette, transferred to 3 cryovials (~2 ml each) and stored at −80 °C. The buffy layer of PBMCs was pipetted into a 15 ml Falcon tube and washed with ice-cold 2% FBS/1× PBS by filling the tube then collecting the pellet generated by centrifugation at 500 × *g* for 8 min at 3 °C. The supernatant was discarded and the PBMC pellet was resuspended in 500 μl of ice-cold 1 × PBS, aliquoted equally into 3x cryovials and stored in liquid nitrogen.

### Measurement of markers of hepatic and renal function

The activity of alanine aminotransferase and alkaline phosphatase and the concentration of albumin was measured in an aliquot of all plasma samples to indicate liver and kidney function by Jinfiniti Precision Medicine, Augusta, USA.

### Measurement of NAD^+^ and selected, related metabolites

NAD^+^ in whole blood was measured using 2 techniques – a commercial kit that detects total NAD^+^ plus NADH as a luminescent signal as luciferin is generated from pro-luciferin by an NADH-dependent reaction (NAD^+^/NADH-Glo Assay, Promega), and also, along with nicotinamide mononucleotide (NMN) and nicotinamide (NAM), by LC–MS. Measurement using the NAD^+^/NADH-Glo Assay was in accordance with the manufacturer’s instructions. Measurement by LC–MS was through a commercial service provided by Northumbria University, Newcastle upon Tyne, UK. Samples were prepared by addition of 13C tryptophan as an internal standard to 100 μl whole blood, followed by 700 μl 2:1 chloroform/methanol and, after sonication on ice, 400 μl LC/MS grade water. Following further sonication on ice, the upper layer separated by centrifugation was dried down under vacuum and reconstituted in 75 μl LC/MS grade water then filtered through a 0.22 μm column. Separation by LC was on a Waters Acquity UPLC BEH amide column (2.1 × 150 mm with particle size of 1.7 μm) at 65 °C using a binary buffer system: 95%/5% (LC/MS grade water/acetonitrile) and 90/10% (acetonitrile/water). MS data were acquired using a Q Exactive Hybrid Quadrupole-Orbitrap mass spectrometer (Thermo Scientific) with orbitrap detector operating parameters of: mass resolution 30 K; mass scan range 100–700 m/z; RF lens 35%; normalised AGC target 25% (100% = 3e6); intensity threshold 2e4. All data were acquired in profile mode.

### Western blotting

Western blotting was used to measure SIRT1 (mouse monoclonal antibody, Cat. No. 60303-1-Ig, Proteintech), NAMPT (rabbit polyclonal antibody, Cat. No. 11776-1-AP, Proteintech), PGC1α (mouse monoclonal antibody, Cat. No. 66369-1-Ig, Proteintech) and CD38 (mouse monoclonal antibody, Cat. No. 60006-1-Ig, Proteintech) in PBMCs, relative to ACTB as loading and transfer standard (mouse monoclonal antibody, Cat. No. 60008-1-Ig, Proteintech). Samples (10 μg protein, quantified using a BCA assay (Micro BCA, Pierce) and Bovine serum albumin (BSA) as a series of standards) were separated by electrophoresis through a 10% polyacrylamide gel then transferred to PDVF membrane using the wet transfer method. Tris-buffered saline, containing 0.15 Tween (TBST) and 5% BSA or, for NAMPT, 5% milk powder, was used for blocking (45–60 min at room temperature), incubation with specific primary antibody (all used at 1:500 dilution, 4 °C overnight), incubation with ACTB antibody (1:10,000, 4 °C overnight) and for incubation with secondary antibody (Polyclonal Goat Anti-Mouse Immunoglobulin/HRP, Cat. No. P044701-2, Dako or, for NAMPT, Polyclonal Goat Anti-Rabbit Immunoglobulin/HRP, Cat. No. P044801-2, Dako) used at 1:2000, except to detect the ACTB primary antibody for which it was used at 1:5000, 1 h at room temperature. Washing between incubations was in TBST. Supplementary Table 5 summarises all antibody sources and dilutions. Secondary antibody was detected using ECL plus or ECL regular (Pierce) and a Syngene G:BOX system. Images were saved as TIF files then densitometry measurements were made using ImageJ software. Band intensities were expressed relative to the corresponding sample intensity on the same blot for ACTB and, to enable pooling of data across different blots for statistical analysis, then normalised to the mean of the corresponding baseline (pre-supplement) measures (relative to ACTB).

### Measurement of cytokines in plasma

Selected cytokines were measured in plasma as a service provided by Eve Technologies, Calgary, Canada using the Human High Sensitivity T-Cell 14-plex Discovery Assay® Array (HDHSTC14).

### Measurement of glycated serum protein

Levels of serum protein glycation in plasma samples at baseline and 28 days for both arms of the trial was measured by Jinfiniti Precision Medicine, Augusta, USA.

### Measurement of biological age using IgG glycosylation profiling

Changes to IgG glycosylation patterns were used as a measure of ‘biological age’ in plasma samples at baseline and 28 days for both arms of the trial, using a commercial service provided by Glycanage Ltd., UK.

### Statistical analysis

Measurements of NAD^+^, NAM and NMN in whole blood (multiple time points) were expressed as difference from the mean of the 2 baseline measurements and analysed using repeated measures 2-way ANOVA, followed by Bonferroni post-hoc test (SPSS). Measurements of cytokines in plasma at 28 days were expressed as difference from the mean of the 2 baseline measurements and analysed by Student’s paired *t*-test. Western blotting densitometric data for SIRT1, NAMPT, PGC1α and CD38 was normalised to ACTB, then expressed as a percentage of the mean of the baseline measurements and analysed by Student’s *t*-test. Measurements of IgG glycosylation and serum protein was analysed by Student’s paired *t*-test.

### Reporting summary

Further information on research design is available in the Nature Research Reporting Summary linked to this article.

### Supplementary information

Supplementary information

Reporting summary

## Data Availability

The datasets used and/or analysed during the current study available from the corresponding author on reasonable request.
