# Supplementary material for: The use of a systems approach to increase NAD+ in human participants
Source: NPJ Aging. 2024 Feb 1;10(1):7. doi: 10.1038/s41514-023-00134-0 (PMC10834541; doi:10.1038/s41514-023-00134-0)
Supplement: Supplementary file 1 — Supplementary information [file 41514_2023_134_MOESM1_ESM.pdf]

Supplementary information

Supplementary Table 1. Participant ages and genders, showing which of the 9 different measures made in the study were on samples from each participant.

Participant no.	Age	Gender	NAD^+ Assay (n=23)	NAD^+ LC-MS (n=17)	Cytokine Assay (n=16)	Protein Glycation (n=24)	IgG Glycosylation (n=24)	WB – NAMPT (n=21)	WB - SIRT1 (n=18)	WB - PGC1α (n=17)	WB - CD38 (n=19)
1	42	Female				yes	yes	yes	yes		
2	72	Female	Yes	yes		yes	yes				
3	34	Female	Yes	yes							
4	29	Female	Yes	yes	yes	yes	yes	yes			yes
5	21	Female			yes	yes	yes	yes	yes		
7	62	Female	Yes	yes		yes	yes	yes	yes	yes	yes
9	51	Female	Yes	yes		yes	yes	yes	yes	yes	yes
10	53	Female	Yes	yes	yes	yes	yes				
11	40	Female	Yes	yes	yes	yes	yes	yes	yes	yes	yes
12	43	Female	Yes	yes		yes	yes	yes	yes	yes	yes
14	39	Female	Yes	yes	yes	yes	yes	yes	yes	yes	yes
15	41	Female	Yes	yes	yes	yes	yes	yes	yes	yes	yes
17	59	Female	Yes		yes	yes	yes	yes	yes	yes	yes
18	22	Female	Yes	yes	yes	yes	yes	yes	yes	yes	yes
19	52	Female	Yes			yes	yes				
20	60	Female	Yes		yes	yes	yes	yes	yes	yes	yes
21	49	Female	Yes	yes	yes	yes	yes	yes	yes	yes	yes
22	23	Male	Yes		yes	yes	yes	yes	yes	yes	yes
23	61	Male	Yes	yes	yes	yes	yes	yes	yes	yes	yes
24	44	Male	Yes	yes	yes	yes	yes	yes	yes	yes	yes
25	60	Female	Yes	yes		yes	yes	yes	yes		yes
26	66	Male	Yes		yes	yes	yes	yes	yes	yes	yes
27	68	Male	Yes	yes		yes	yes	yes		yes	yes
28	62	Male	Yes		yes	yes	yes	yes	yes	yes	yes
29	39	Male	Yes	yes	yes	yes	yes	yes	yes	yes	yes

Supplementary Table 2. Age profile and gender split of study participants

Participants who completed the study	25
Average Age	48
Median Age	49
20-39 Age group	7 (28%)
40-59 Age group	10 (40%)
60+ Age group	8 (32%)
Total Females	17 (72%)
Total Males	8 (28%)

Supplementary Table 3. Adverse effects reported by study participants.

Major Adverse Events	Minor Adverse Events	Condition	N
none	Stomach Irritation	NT	1 (4%)
	Trouble Sleeping	NT	2 (8%)
	Hyperactivity	placebo	1 (4%)

Supplementary Table 4. Comparison by participant of the measured change in NAD^+ concentration in whole blood from baseline, expressed as a percentage, by enzymatic assay and LC-MS.

Participant no.	Age	Gender	Change from baseline by enzymatic assay (%)				Change from baseline by LC-MS (%)			
			Placebo Week 3	Placebo Week 4	NT Week 3	NT Week 4	Placebo Week 3	Placebo Week 4	NT Week 3	NT Week 4
2	72	F	102.22	98.15	120.87	103.43	89.48	67.05	122.82	187.39
3	34	F	110.06	109.24	103.05	131.76	131.21	134.88	182.52	174.39
4	29	F	107.00	111.19	90.21	84.89	160.91	148.76	97.74	159.15
7	62	F	88.57	107.45	136.68	138.67	81.73	88.36	79.89	83.63
9	51	F	86.97	97.74	124.10	131.08	85.12	58.00	144.35	252.89
10	53	F	99.13	103.08	124.97	142.26	97.08	81.09	79.37	48.91
11	40	F	96.81	105.99	165.61	165.15	138.42	121.57	117.65	125.93
12	43	F	93.50	100.27	113.41	106.37	138.12	137.05	85.87	83.92
14	39	F	103.72	106.10	120.39	124.85	119.62	113.65	94.27	89.64
15	41	F	51.37	56.30	125.37	112.90	131.45	118.84	123.74	137.61
18	22	F	101.98	105.03	116.74	165.54	117.05	127.77	118.48	177.55
21	49	F	101.61	99.93	118.35	108.39	98.17	128.96	148.41	161.90
23	61	M	123.42	133.59	203.57	162.55	57.51	76.31	114.88	116.93
24	44	M	97.97	97.21	87.95	78.69	111.04	98.79	110.85	112.52
25	60	F	105.89	112.76	115.57	107.82	187.34	115.46	106.25	93.72
27	68	M	104.88	119.71	88.82	92.54	105.15	102.33	112.28	101.16
29	39	M	81.87	86.05	92.82	105.11	107.13	97.83	80.79	97.76

Supplementary Table 5. Antibody sources and dilutions.

Primary antibodies		
**Target antigen**	**Supplier and catalogue number**	**Dilution**
SIRT1	Proteintech, Cat. No. 60303-1-Ig,	1:500 in TBST; 5% skimmed milk
NAMPT	Proteintech, Cat. No. 11776-1-AP	1:500 in TBST; 5% skimmed milk
PGC1α	Proteintech, Cat. No. 66369-1-Ig	1:500 in TBST; 5% skimmed milk
CD38	Proteintech, Cat. No. 60006-1-Ig	1:500 in TBST; 5% skimmed milk
ACTB	Proteintech, Cat. No. 60008-1-Ig	1:10,000 in TBST; 5% skimmed milk
**Secondary antibodies**		
**Conjugate**	**Supplier and catalogue number**	**Dilution**
Horseradish peroxidase (HRP)	[1]Dako, Cat. No. P044701-2	[3]1:2000 or [4]1:5000
Horseradish peroxidase (HRP)	[2]Dako, Cat. No. P044801-2	1:2000

[1]For detection of all primary antibodies except NAMPT
[2]For detection of NAMPT primary antibody
[3]For detection of all primary antibodies except ACTB
[4]For detection of ACTB primary antibody

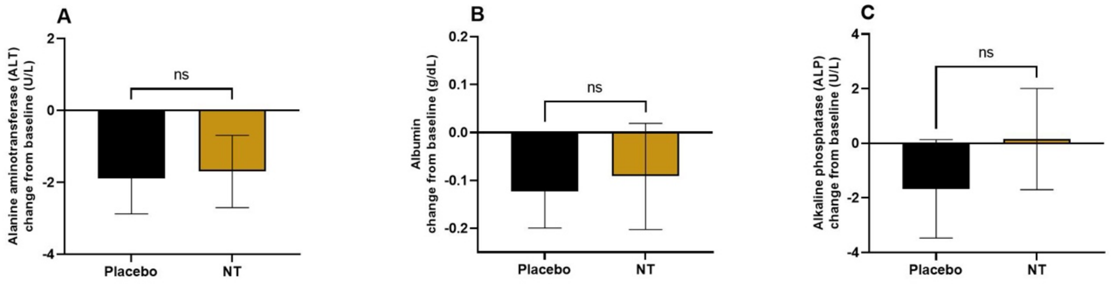

Supplementary Figure 1. Measurements of alanine aminotransferase and alkaline phosphatase and the concentration of albumin in plasma samples after 28 d on placebo or NT. Data are shown relative to baseline as mean ± SEM, n=24. NS – not significant (P>0.05) by Student's paired t-test.

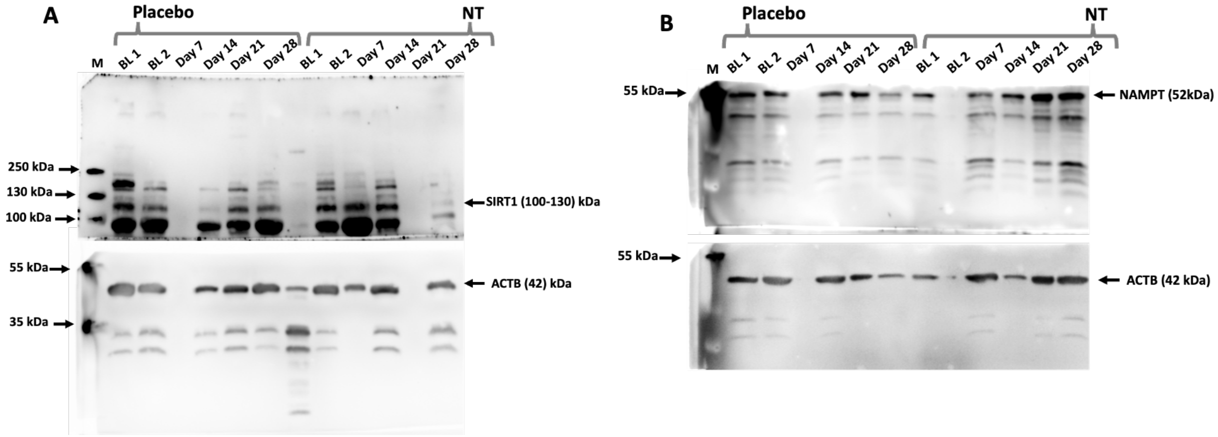

Supplementary Figure 2. Uncropped blots for SIRT1 (Panel A) and NAMPT (Panel B), and corresponding ACTB loading controls. Samples are as labelled. M – molecular weight marker (PageRuler; 10-250kDa)
